# Environmental determinants of phylogenetic diversity in vernal pool habitats

**DOI:** 10.1002/ece3.11583

**Published:** 2024-06-24

**Authors:** Brandon Hendrickson

**Affiliations:** ^1^ University of Louisiana Lafayette Louisiana USA; ^2^ University of California Merced California USA

**Keywords:** community turnover, competition relatedness hypothesis, habitat filtering, horizontal beta diversity, phylogenetic structure

## Abstract

Phylogenetic diversity offers critical insights into the ecological dynamics shaping species composition and ecosystem function, thereby informing conservation strategies. Despite its recognized importance in ecosystem management, the assessment of phylogenetic diversity in endangered habitats, such as vernal pools, remains limited. Vernal pools, characterized by cyclical inundation and unique plant communities, present an ideal system for investigating the interplay between ecological factors and phylogenetic structure. This study aims to characterize the phylogenetic patterns of vernal pools and their associated vegetation zones, addressing questions about taxonomic and phylogenetic community discreteness, the role of flooding as a habitat filter, the influence of invasive species on phylogenetic structure, and the impact of seasonal variation on phylogenetic diversity. I find that zones‐of‐vegetation exhibit high between zone taxonomic and phylogenetic beta diversity whereas each zone forms a unique cluster, suggesting that zones are taxonomically and phylogenetically discrete units. Regions of high‐inundation pressure exhibit phylogenetic clustering, indicating that flooding is a habitat filter in vernal pool habitats. Competition between native species conform to the ‘competitive relatedness hypothesis’ and, conversely, communities dominated by invasive Eurasian grass species are phylogenetically clustered. In addition, I find that phylogenetic diversity within zones fluctuates across the spring season in response to changing water levels, precipitation, and temperature. By analyzing three pools within the Merced Vernal Pool and Grassland Reserve, this research elucidates the phylogenetic dynamics of vernal pools. The findings underscore the need for tailored conservation strategies that account for the unique ecological characteristics of each vegetation zone within vernal pool habitats.

## Graphical abstract

The present article investigates two contrasting hypotheses of competition on community phylogenetic structure, finding evidence for both though outcomes are contingent upon invasion intensity. In addition, seasonal climate variance is found to drive changes in phylogenetic diversity over time in accordance with environmental stressors such as inundation. The present article provides a new framework of understanding the phylogenetic consequences of invasion on native communities and highlights the temporal dynamics of phylogenetic diversity in plant communities.

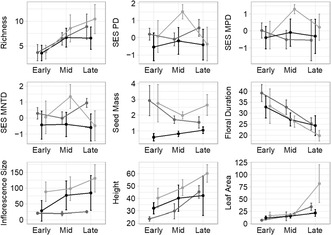

## 1 INTRODUCTION

Phylogenetic diversity gives insight into the underlying ecological factors that influence species composition and, in turn, can be used to direct conservational management. Plant phylogenetic diversity unites niche preferences and traits and is a reliable proxy of vital ecosystem functions (Srivastava et al., 2012), community productivity (Cadotte et al., 2009) and community stability (Cadotte et al., 2012). Combined with taxonomic diversity, phylogenetic diversity can be used to predict the multifunctionality of ecosystems. Therefore, rehabilitation of endangered habitats necessitates the analyses of phylogenetic diversity (Barak et al., 2017; Barber et al., 2017; Larkin et al., 2015), however, this practice has not been universally adopted.

Vernal pools are ephemerally aquatic habitats underlain by an impermeable claypan that promotes the cyclical filling of water during the winter and evaporation during the spring (Keeley & Zedler, 1998; Smith & Verrill, 1998; Solomeshch et al., 2007). Approximately 95% of vernal pool habitats in the Central Valley of California have been destroyed (Ruiz‐Ramos et al., 2023) and continue to be threatened by altered climate and persistent invasions. To date, no phylogenetic diversity assessment has been performed on the habitat, and thus, the ecological factors that maintain phylogenetic diversity and the human impacts that disrupt phylogenetic structure are not currently understood.

Vernal pool environments are not only marked by predictable cycles of flooding, but also by unique communities of plants that form concentric bands along the slope of each pool called zones‐of‐vegetation. Heterogeneous vegetation zones are formed by inundation gradients that select for species able to tolerate a spectrum of flooding pressure (Barbour et al., 2003; Deil, 2005; Gosejohan et al., 2017; Solomeshch et al., 2007). The inundation gradient also limits invasion primarily to the upper portions of a pool though several invasives do inhabit the pool bottom (Bauder, 2000; Gerhardt & Collinge, 2003, 2007). High dissimilarity between zones‐of‐vegetation in combination with low‐beta diversity among zones has been used to confirm the existence of these taxonomically discrete units (Michaels et al., 2021). However, phylogenetic diversity of each zone‐of‐vegetation has not been characterized due to previously unresolved species relationships. With recently constructed megaphylogenies that include vernal pool families (Smith & Brown, 2018), several aspects of vernal pools can be analyzed in a phylogenetic framework.

First, the effect of inundation on phylogenetic structuring across zones‐of‐vegetation can be analyzed. Inundation effectively controls species distributions in accordance with physiological tolerances (Bauder, 2000; Emery et al., 2009; Holland & Jain, 1988; Schlising & Sanders, 1982). Habitats characterized by steep environmental stress gradients select for communities of species based on functional traits (Cornelissen et al., 2003; Mouillot et al., 2012), and result in phylogenetically nonrandom communities (Webb et al., 2002). Environmental filtering on phylogenetically conserved traits can produce co‐occurrence of closely related species (Bartish et al., 2010; Gerhold et al., 2008; Kembel & Hubbell, 2006; Webb, 2000). The exclusion of species unable to tolerate stressful abiotic factors has been coined as “habitat filtering” (Diaz et al., 1999) and has been classically associated with phylogenetic clustering (Cahill et al., 2008; Gerhold et al., 2015; Lososová et al., 2015; Price & Pärtel, 2013). Therefore, inundation in vernal pools may result in phylogenetic distinct communities that parallel taxonomically defined ‘zones‐of‐vegetation’ as well as produce a gradient of phylogenetically clustered communities in successively more inundated regions.

Second, the level of invasion varies across zones‐of‐vegetation (Bauder, 2000; Gerhardt & Collinge, 2003, 2007) that may prove amenable for assessing the effect of competition on phylogenetic diversity in vernal pools. Invasive species exert competitive pressure on native plant communities that in turn can lead to altered taxonomic and phylogenetic diversity (Castellani et al., 2022; Lishawa et al., 2019). In the past four centuries of European colonization into North America, thousands of plant species have been introduced and hundreds have become invasive in their new range (Hierro et al., 2005; Pyšek et al., 2015). Although approximately 1 in 10 introduced species are invasive (Holgate, 1986; Williamson & Brown, 1986), the species that successfully spread often do so by outcompeting natives for resources and space (Gurevitch & Padilla, 2004; Michelan et al., 2018; Sax & Gaines, 2008). Loss of native biodiversity and consistent introduction of competitive invasives can fundamentally alter the phylogenetic structure of a community by either promoting phylogenetic clustering when nonnatives originate from the same location and exhibit a phylogenetic signal of competitiveness (Mayfield & Levine, 2010) or by leading to phylogenetic overdispersion if competition occurs between closely related native and nonnative species (Macarthur & Levins, 1967; Mouquet et al., 2012; Webb et al., 2002).

The exact phylogenetic outcome of invasion – clustering or overdispersion – is of conservational importance to vernal pools as phylogenetically clustered communities are more invadable due to emptier niche space (Lososová et al., 2015). If competitive invasives drive phylogenetic clustering in vernal pool habitats, such a change can facilitate a positive feedback cycle of further invasion. Furthermore, if phylogenetic clustering is associated with reduced functional diversity, ecosystem functionality and services may be threatened leading to instability. Therefore, understanding the impact of contemporary invasions on vernal pool zones‐of‐vegetation can provide valuable insight into the future threat of biotic invasions and stability of the habitat.

Finally, each zone‐of‐vegetation exhibits a steep temporal gradient of water availability, particularly at the pool bottom and edge, which are strongly associated with species turnover during the growing season (Rosario, 1979). Thus, there is a question of how phylogenetic diversity changes over time in vernal pool habitats. Fluctuating environmental conditions select for unique plant assemblages that capitalize on optimal conditions at different times (Tonkin et al., 2017). A corollary of this phenomenon is that shared physiological tolerances lead to overlapping species presence in space and time (Mathias & Chesson, 2013). As such, conserved traits for niche specialization can result in changes to phylogenetic diversity as unique communities emerge in response to shifting seasonal conditions (Strauß et al., 2016; Yan et al., 2023). Vernal pool habitats predictably shift from inundated wetlands to desiccated deserts over several months, which fosters the transition from wetland adapted species to mesic adapted species and, finally, to xeric adapted species. If niche preferences are highly conserved among vernal pool plant communities, species turnover in response to changing water levels may be accompanied by changes in phylogenetic diversity.

In this study, the objective is to characterize the phylogenetic patterns of vernal pools and the zones‐of‐vegetation by addressing the following questions: Are vernal pool zones‐of‐vegetation taxonomically and phylogenetically discrete units?, Does flooding act as a habitat filter indicated by phylogenetic clustering?, Does competition by invasive species influence phylogenetic structure?, and Does seasonal variation throughout spring drive changes in phylogenetic diversity? I utilize 3 pools located on the Merced Vernal Pool and Grassland Reserve at the University of California, Merced as the study system. The results of these tests are used to identify the phylogenetic dynamics of vernal pools, an endangered habitat, in response to the presence of predictable flooding, seasonal water cycles, and biotic invasions. Effective conservation of vernal pool habitats must consider each zone‐of‐vegetation uniquely to maintain ecosystem functionality. To do so, understanding the phylogenetic diversity and structure of each zone can provide information regarding its ecology and give necessary context to the conservational challenges facing each community.

## 2 METHODS

### 2.1 Study site

The San Joaquin vernal pool megacomplex located in Merced, CA and managed by the University of California Reserve System is one of the largest actively preserved vernal pool environments in the Western United States. The megacomplex exists along the edge of the hot Mediterranean and cold semi‐arid climate regime defined by Koppen, characterized by cold inclement winters, hot desiccating summers, and high interannual climate variability (Kesseli, 1942). Pools ranging from an area of 200–600 square meters and a depth of <0.5 m were chosen for observation, which are representative of most pools at the study site (B. Hendrickson *pers. obs*.).

### 2.2 Climate sampling

Climate monitoring of the Central Valley of California is performed by the California Irrigation and Management Information System (CIMIS), which is tasked with monitoring biologically informative climate variables used to make management decisions for farmland and cultivation. The CIMIS climate station located approximately 10 km from the Merced Vernal Pool and Grassland Reserve was used as the source of climate data.

Daily precipitation recordings from October 1st to September 31st from 2018 to 2022 and 3 primary temperature metrics from the CIMIS climate station were used: maximum daily temperature, minimum daily temperature, and average daily temperature. Using daily precipitation volume, cumulative rainfall from October 1st to September 31st for each year was calculated.

### 2.3 Species sampling

Species observations were taken on a weekly basis from March (3/1) to May (5/1) at the three observation pools, corresponding to the greatest biological activity. The sampling survey ended when all plants in the habitat had senesced which differed between years. Thus, the number of sampling periods differed between years: 7 in 2019, 8 in 2020, and 9 in 2021. Observations of each pool were conducted at the same time of day (11 am–2 pm) each week. Weekly observations of species presence for each pool were made for approximately 45 minutes, devoting equal time to each zone of the pool.

Species were identified by employing the Jepson Manual of California species (Greenhouse, 2012). Following the dichotomous key of the manual, morphological and reproductive traits were used to identify plants at the species level. Floral traits were often the distinguishing factor between closely related taxa due to the petite stature of most vernal pool species and the similarity of foliage across taxa. Consequently, species identities were made when the plant was flowering. The timing of the first flowering was recorded and then monitored each week. A caveat is that plants in the vegetative stage could not be identified despite potentially imparting competitive effects on surrounding vegetation.

### 2.4 Defining zones by abiotic gradients

Prior research on vernal pool communities strongly suggests that inundation and clay content are the two major environmental factors differentiating plant communities (Bauder, 2005; Deil, 2005; Gosejohan et al., 2017). Gosejohan et al. (2017) extensively sampled the plant community and abiotic environmental factors of two high‐elevation vernal pools at Modoc Plateau, CA, discovering (1) The presence of three clearly defined vernal pool zones distinguished primarily upon inundation length, and (2) No influence of any other measured variable on community composition (total herbaceous cover, coarse woody debris cover, livestock hoof print cover, and livestock scat cover). The presence of ‘zones‐of‐vegetation’ has neither been confirmed nor characterized for the San Joaquin vernal pool megacomplex. In this study, zones are distinguished along two environmental axes: inundation and clay content, which are both associated with niche adaptation of terrestrial and aquatic species (Blom, 1999; Rajakaruna, 2018), thus providing a foundation for segregating communities of plants.

North–south and east–west transects spanning each pool and approximately 2 m into the grassland were drawn each week at the same position in the pool. Sizes of the pools are summarized in Table 1. Because soil cannot be removed from the pool as per guidelines for preserving rare animal and plant species that have cysts and seeds in the soil, in situ, clay content was assessed using the ‘ribbon method’ (Thien, 1979). The clay content of each pool was sampled 5 times every meter along the transect on September 20, 2019. Given that clay was sampled once, it cannot be assumed that clay content is stable across years. Soil types were easily distinguishable by both touch and appearance, which paralleled observed changes in clay content (B. Hendrickson *pers. obs*.). In addition, the length of inundation for each pool was tracked each year. A pool was considered “dry” when no standing water was present.

**TABLE 1:** Dimensional and edaphic characteristics of three observation pools on the Merced Vernal Pool and Grassland Reserve.

Pool	N–S distance (m)	E–W distance (m)	Area (m^2^)	Max depth (m)	Estimate volume (m^3^)	Clay bottom %	USGS soil type
1	13.10	21.50	281.65	0.47	34.66	83.60	ReB
2	13.30	13.70	182.21	0.24	11.45	78.40	CkB
3	19.10	19.90	380.09	0.31	30.85	75.20	CkB

*Note*: North–South (N–S) and East–West (E–W) distances were measured using a transect spanning the pool bottom and 2 m into the pool upland. The pool area was calculated as the elliptical area of the pool. Max depth was the height of water in the center of a pool during the wettest year and while the pool was inundated. Estimate volume was calculated by using the maximum depth of water and the elliptical area of the pool to determine the hemispheric volume. Clay percentage is the average clay content found throughout the bottom of each pool. Soil types described by the USGS soil survey are Redding gravelly loam with 0 to 8 percent slopes (ReB) and Corning gravelly sandy loam with 0 to 8 percent slopes (CkB).

Clay content was used primarily to distinguish between the pool bottom and edge zones. To distinguish between the pool edge and grassland (hereafter referred to as the upland), the water level during peak inundation was marked along the transect. After determining where the zones were in each pool, I then combined the zonal information with the meter distances of inundation to determine the total length of inundation for each zone.

### 2.5 Confirming zones of vegetation based on taxonomic discreteness

**FIGURE 1:**
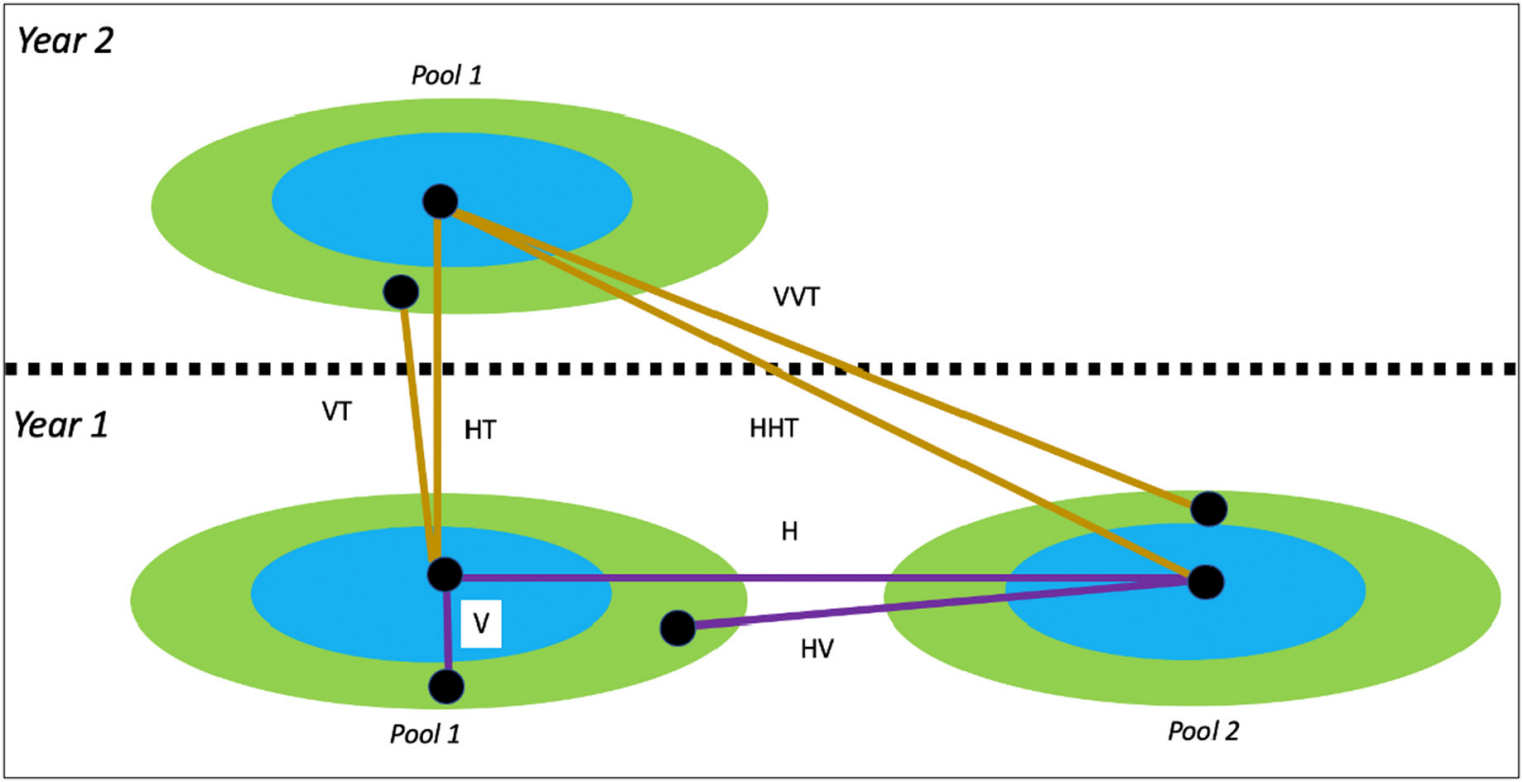
Diagram of vertical and horizontal comparisons across zones of vegetation and pools. H, horizontal beta diversity, compares the same zone of vegetation between two pools within the same year. V compares two zones of vegetation within the same pool and year, conventionally called vertical beta diversity. Simply, a greater vertical beta diversity than horizontal beta diversity suggests vegetation zones are taxonomically discrete. HV compares two zones of vegetation belonging to two different pools in the same year. HV is a measure of vertical beta diversity that includes the influence of geographic distance on community differences. VT compares two zones of vegetation in the same pool between 2 years. HT compares the same zone of vegetation in the same pool between 2 years. Both VT and HT incorporate the potential for annual community turnover to exacerbate or lessen community dissimilarity. HHT compares the same zone of vegetation between two different pools over 2 years. VVT compares two vegetation zones between pools and between years. Both VVT and HHT include geographic and temporal distance as a possible driver of community dissimilarity.

To determine if the zones defined by abiotic factors correspond to taxonomically distinct communities, two community dissimilarity metrics were recorded: Jaccard dissimilarity index and Sorensen's dissimilarity index. Previous studies on vernal pool zones‐of‐vegetation utilized a zone and pool comparison heuristic (Figure 1), which includes comparing two zones of the same pool (vertical beta diversity; hereafter referred to as “V”) and comparing the same zone of two pools (horizontal beta diversity; hereafter referred to as “H”) (Michaels et al., 2021). Statistically lower vertical beta diversity compared to horizontal beta diversity indicates the absence of unique vegetation zones. Alternatively, statistically lower horizontal beta diversity compared to vertical beta diversity means that distinct communities have been established along the slope of a pool indicating the presence of true zones‐of‐vegetation. One way analysis of variance was conducted with vertical and horizontal comparison classes as categorical predictor variables and beta diversity as the response variable. Analysis of variance (ANOVA) was conducted with the car package (Fox & Weisberg, 2019) in R. NDMS was performed on zones to further confirm the discreteness of zones and provide consilience using the vegan package in R (Oksanen et al., 2022).

I expand upon previous studies by testing multiple ecological predictions concerning the influence of temporal and spatial distance on community structure. The various comparisons and their respective ecological interpretation are as follows. Vertical beta diversity between pools (VH) is defined as taxonomic dissimilarity between different zones of two pools. This measure incorporates geographic distance as a potential factor differentiating two zones from each other. The first metric that incorporates interannual community variation is HT, defined as beta diversity of the same zone within a pool between years. HT incorporates the potential for climate factors to interact with a diverse seed bank within a pool to produce significantly different communities. HHT is beta diversity of the same zone between 2 years and two different pools. HHT incorporates the same climate factors as HT but also includes geographic distance as an additional differentiation factor. HHT presumes that the communities inhabiting the same zone of two different pools may be influenced by priority affects and dispersal limitation. There were two temporal vertical beta diversity measures; the first was community dissimilarity of different zones within the same pool between years (VT), and the second was community dissimilarity of different zones between two different pools and years (VVT). Significant differences between comparison classes were determined using ANOVA and Tukey HSD in the car (Fox & Weisberg, 2019) and stats packages, respectively, the latter being part of base R (R Core Team, 2021).

### 2.6 Phylogeny construction

Using 42 species recorded across all pools and years, a phylogeny was constructed using the R package V.PhyloMaker2 (Jin & Qian, 2022). The package uses a dataset of resolved relationships from 75,000+ species covering all extant vascular families (Smith & Brown, 2018). The species names of vernal pool plants were updated using the Catalog of Life checklist prior to assembling the tree. The synthesis phylogeny contains molecular branch lengths and is scaled to time so that phylogenetic distance can be calculated.

### 2.7 Phylogenetic discreteness of vegetation zones

To assess if each zonal community forms a unique phylogenetic community, among‐community phylogenetic distance (i.e., phylogenetic beta diversity) was calculated. A dendrogram was then constructed to visualize clusters of zones across the three observation pools. A monophyletic cluster for each zone indicates that zones‐of‐vegetation are phylogenetically distinct. Phylogenetic beta diversity was calculated using the picante package in R (Kembel et al., 2010), and hierarchical clustering was performed using the stats package of base R (R Core Team, 2021). Furthermore, it is known that taxonomic beta diversity between vernal pool regions is high (Buck, 2004), though no comparison of phylogenetic diversity has been performed between vernal pool regions to date. To assess how phylogenetic diversity is distributed across vernal pool regions of California, I analyzed the phylogenetic beta diversity of two vernal pools described by Gosejohan et al. (2017) found at the Modoc Plateu, a high‐altitude vernal pool region in Northern California, in concert with three vernal pools sampled in Merced, California. This was performed to understand how zones‐of‐vegetation are conserved across space and, in turn, inform how conservational practices may need to be regionally dependent.

### 2.8 Phylogenetic diversity of vegetation zones

Phylogenetic distance and community matrix construction were performed using the picante package of R (Kembel et al., 2010). The phylogenetic distance and community data matrices were used to calculate Faith's phylogenetic distance (PD), mean nearest taxon distance (MNTD), and mean phylogenetic distance (MPD). Phylogenetic diversity values calculated by synthesis phylogenies are strongly correlated with phylogenies constructed from gene sequence data (Allen et al., 2019; Jantzen et al., 2019; Li et al., 2019). PD_Faith_ values were calculated using the picante package in R (Kembel et al., 2010). Faith's PD is biased by species richness; thus, I calculated the standardized effect size to control for this. MPD is used to determine tree‐wide patterns of phylogenetic structure (Webb et al., 2002). To assess patterns of phylogenetic structure attributable to the phylogenetic distance of closely related taxa, the mean nearest taxon distance was calculated (Webb et al., 2002). Standardized effect sizes of PD_Faith_, MNTD, and MPD were calculated using the picante package of R (Kembel et al., 2010).

To assess the phylogenetic consequences of habitat filtering on plant communities, linear regression models for each zone were constructed with inundation length as the predictor variable and MNTD or MPD as response variables. A positive or negative slope would indicate that greater inundation leads to phylogenetic overdispersion or clustering, respectively. Linear regressions were performed using the stats package in base R (R Core Team, 2021).

### 2.9 Trait sampling

Two classes of functional traits were sampled: morphological and phenological. Phenological traits include mean first flowering date, mean floral termination date, and mean floral duration. These traits were collected by monitoring the floral schedule of each plant in the field. Morphological traits include inflorescence size, leaf area, seed mass, and height. All morphological traits were recorded from the Jepson Manual of California Species (Greenhouse, 2012). For species that were not included in the Jepson Manual of California Species, online databases such as “Gobotany.com” were used. Several species attributes were recorded such as if the plant was an invasive, if the plant was a nitrifier, and entomophily or anemophily.

### 2.10 Quantifying competitiveness on phylogenetic structure

Phylogenetic clustering and overdispersion patterns of different zones were investigated by quantifying the relationship of MPD and MNTD with various environmental drivers – competition or inundation. Competition level for a community was defined as the community‐wide mean of height (CWM.H) and community‐wide mean of leaf area (CWM.LA), two traits that are associated with greater competitive ability in vernal pool plants (Kraft, 2016). Competitiveness was treated as the first principal component axis of CWM.LA and CWM.H. Linear regression models for each zone were created using the PC of competitiveness as a dependent variable with either MPD or MNTD as predictor variables. A positive slope indicates that higher competition is associated with phylogenetic overdispersion, whereas a negative slope indicates that competition is associated with phylogenetic clustering.

Given that higher competition could be associated with either a greater number of highly competitive species or be due to one highly competitive species that skews the mean higher, linear regressions on the first principal component axis of community‐wide variance (CWV) were performed. Community‐wide variance will exhibit the opposite slope of the community‐wide mean if the community of plants is composed of highly competitive species. In contrast, parallel slopes of CWM and CWV that would indicate one or a few highly competitive species drive the community‐wide mean.

### 2.11 Quantifying changes to phylogenetic diversity across the spring season

Three‐time categories were created by dividing the 9‐week spring season from March 1st to May 1st into three 3‐week groups, hereafter called the early, mid, and late seasons. In the years 2019 and 2020, flowering species had senesced before May 1; April 17, 2019 and April 24, 2020. Thus, there were 1 and 2 weeks with at least one flowering species in 2019 and 2020, respectively, composing the “late season” time bin.

Polynomial regressions of PD_Faith_ standardized by effect size were performed for both continuous and discrete time (i.e., early, mid, and late season). Three regression models were applied to each zone by weekly PD_Faith_: linear, quadratic, and cubic. For each zone, the model that resulted in the lowest AIC score was determined, and p‐value was calculated to detect significant correlations of time and phylogenetic diversity. The assumption of homoscedasticity and normality of residuals was tested using the Breusch Pagan test and Shapiro–Wilks test of the final model, respectively. Breusch Pagan test and Shapiro Wilks test were performed using the lmtest (Zeileis & Hothorn, 2002) and stats (R Core Team, 2021) package in R, respectively. Faith's PD was calculated for each subseason by zone, year, and pool and then standardized by effect size. A multiple regression model was conducted for each zone with PD_Faith_ as the response variable and zone, season, and zone by season as predictor variables. An ANOVA was conducted to detect significant differences between groups, followed by Tukey's honest significant difference test to determine the pairwise differences between groups. ANOVA and Tukey's HSD were both conducted in the stats package of base R (R Core Team, 2021).

To determine whether seasonal or annual climate variation primarily drives community turnover and phylogenetic diversity, a series of Mantel tests were performed in the vegan package of R (Oksanen et al., 2022). Each mantel test was conducted with 999 permutations. The community distance matrix used in the Mantel test was calculated using the Jaccard dissimilarity method for each weekly community of a whole pool in the vegan package of R (Oksanen et al., 2022). The phylogenetic distance matrix, using PD_Faith_, was calculated using the picante package of R. Environmental distance matrices were calculated using Euclidean distance for annual and seasonal mean air temperature, maximum air temperature, and total precipitation separately with the ecodist package of R (Goslee & Urban, 2007).

The relationship between functional diversity across time and phylogenetic diversity was analyzed in a multiple regression framework. A persistent question is how changes in phylogenetic diversity correlated with changes in functional diversity so as to predict habitat stability and to assess the fidelity of phylogenetic measurements as a proxy of ecosystem functionality. Furthermore, the importance of changing phylogenetic diversity over a season is of conservational interest if functional diversity exhibits parallels changes. I analyze the correlation between phylogenetic diversity with all functional traits measured as well determine if a significant interaction exists between phylogenetic diversity and time on functional diversity.

## 3 RESULTS

### 3.1 Taxonomic patterns of zones of vegetation

**FIGURE 2:**
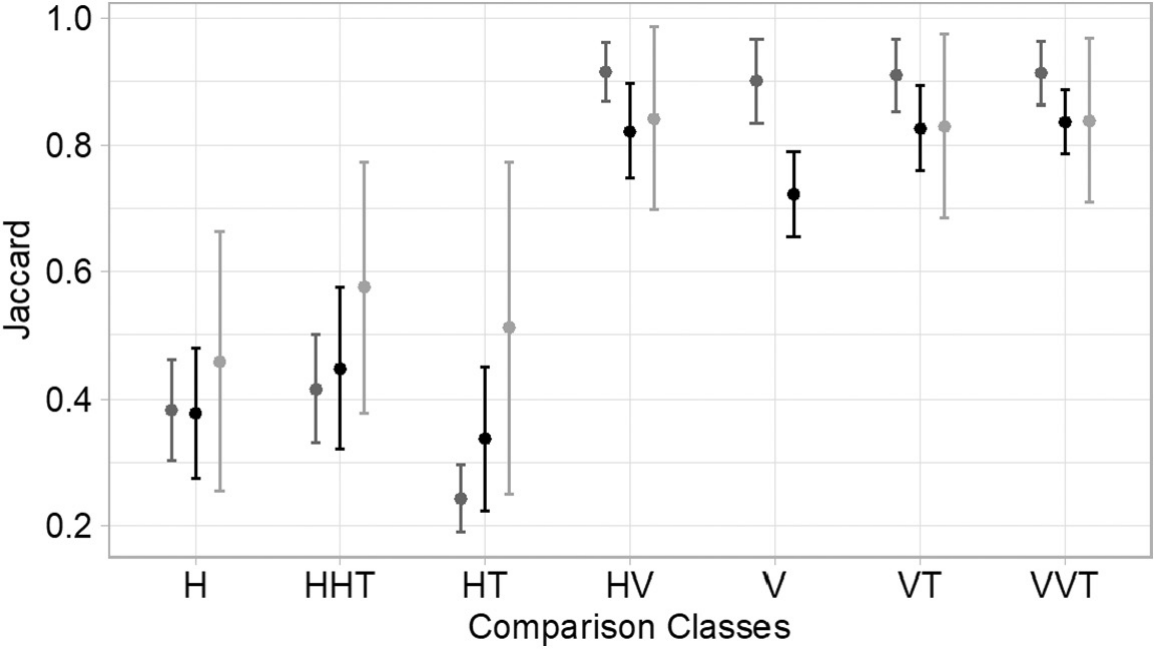
Average horizontal and vertical beta diversity for each zone (light gray: upland, black: edge, and dark gray: bottom) measured as Jaccard dissimilarity. Horizontal beta diversity (H) measures dissimilarity found between the same zone in two different pools (*n* = 9 per zone). HT measures the dissimilarity of a zone of vegetation within the same pool between 2 years (*n* = 9 per zone). HHT measures is the dissimilarity of a zone of vegetation between two pools and 2 years (*n* = 18 per zone). Vertical beta diversity (V) quantifies the community dissimilarity of two zones of vegetation found within the same pool (*n* = 18 per zone). VT measures the dissimilarity between two zones found within the same pool between years (*n* = 18 per zone), and VVT measures the dissimilarity of two zones found within separate pools between years (*n* = 36 per zone). Finally, HV quantifies the dissimilarity of two zones found in separate pools (*n* = 18 per zone).

**FIGURE 3:**
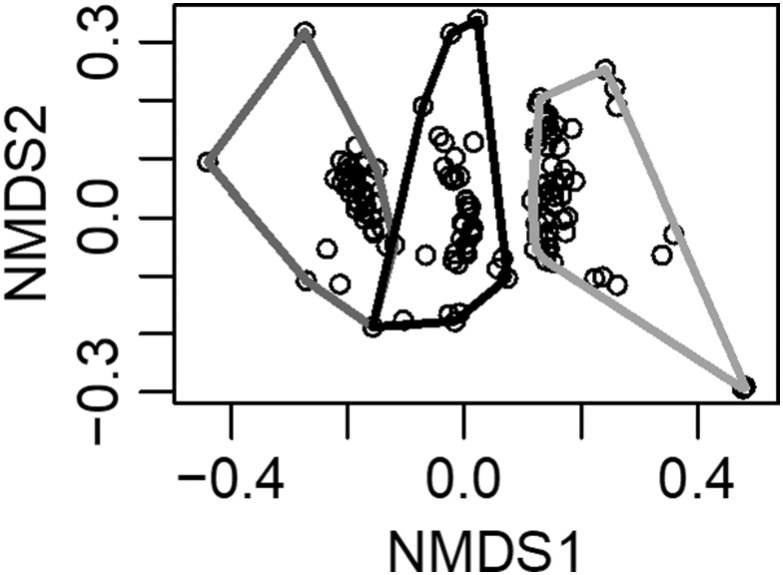
Nonmetric multidimensional scaling of Jaccard distance matrix of weekly vernal pool communities. Convex hulls of pool bottom (dark gray), edge (black), and upland (light gray) vegetation zones are displayed.

Vernal pools in the Central Valley of California have clearly defined zones‐of‐vegetation that parallel the abiotic gradients of inundation and clay content within a pool. Over 3 years in three pools, 42 species were identified to the species level with 30 native and 12 invasive species (Table 2). Taxonomic horizontal beta diversity of vegetation zones was significantly lower than vertical beta diversity, signifying that the same vegetation zone (i.e., upland, edge, bottom) shares more species between pools than different zones within a pool (Figure 2). Furthermore, zones‐of‐vegetation are stable across years, evidenced by a lower HT than H beta diversity (Table 3), which also suggests that geographic distance influences the community composition of a vegetation zone. Regionally, pool bottoms are highly similar to each other across the three pools examined, whereas edge and uplands zones exhibit moderate differences between pools and between years suggesting priority effects and/or higher levels of community turnover (Figure 2). There were no significant differences found among vertical beta diversity metrics (i.e., HV, V, VT, and VVT), indicating that neither time nor space overwhelmed or contributed to differences found between zones‐of‐vegetation. NDMS analyses suggest that bottom, edge, and upland communities exhibit high dissimilarity between zones and relatively low dissimilarity within a zone (Figure 3).

**TABLE 2:** List of native and invasive species found on the Merced Vernal Pool and Grassland Reserve from 2019 to 2021 within the upland (U), edge (E), and bottom (B) zones.

Species	Genus	Family	Native	Zone
*Achyrachaena mollis*	*Achyrachaena*	Asteraceae	Yes	U,E
*Alopecurus pratensis*	*Alopecurus*	Poaceae	No	U
*Amsinckia menziesii*	*Amsinckia*	Boraginaceae	Yes	U
*Avena fatua*	*Avena*	Poaceae	No	U
*Briza minor*	*Briza*	Poaceae	No	U,E
*Brodiaea elegans*	*Brodiaea*	Asparagaceae	Yes	U
*Bromus hordeaceus*	*Bromus*	Poaceae	No	U
*Calandrinia menziesii*	*Calandrinia*	Montiaceae	Yes	U,E
*Capsella bursa‐pastoris*	*Capsella*	Brassicaceae	No	U
*Castilleja attenuata*	*Castilleja*	Orobanchaceae	Yes	U,E
*Cotula coronopifolia*	*Cotula*	Asteraceae	No	E,B
*Deschampsia danthoniodes*	*Deschampsia*	Poaceae	Yes	U
*Dichelostemma capitatus*	*Dichelostemma*	Asparagaceae	Yes	U
*Downingia pulchella*	*Downingia*	Campanulaceae	Yes	B
*Eleocharis macrostachya*	*Eleocharis*	Cyperaceae	Yes	E
*Erodium botrys*	*Erodium*	Geraniaceae	No	U
*Erodium cicutarium*	*Erodium*	Geraniaceae	No	U
*Eryngium aristulatum*	*Eryngium*	Apiaceae	Yes	B
*Eschscholiza lobbii*	*Eschscholiza*	Papaveraceae	Yes	U
*Festuca perennis*	*Festuca*	Poaceae	No	U
*Hesperevax caulescens*	*Hesperevax*	Asteraceae	Yes	B
*Hordeum brachyantherum*	*Hordeum*	Poaceae	No	U
*Hypochaeris glabra*	*Hypochaeris*	Asteraceae	No	B
*Lasthenia fremontii*	*Lasthenia*	Asteraceae	Yes	B
*Lepidium nitidum*	*Lepidium*	Brassicaceae	Yes	U,E
*Limnanthes douglasii*	*Limnanthes*	Limnanthaceae	Yes	E,B
*Mimulus tricolor*	*Mimulus*	Phrymaceae	Yes	B
*Muilla maritima*	*Muilla*	Asparagaceae	Yes	U
*Plagiobothrys humistratus*	*Plagiobothrys*	Boraginaceae	Yes	E,B
*Plagiobothrys nothofulvus*	*Plagiobothrys*	Boraginaceae	Yes	U
*Pogogyne zizyphoroides*	*Pogogyne*	Lamiaceae	Yes	B
*Psilocarphus chilensis*	*Psilocarphus*	Asteraceae	Yes	B
*Psilocarphus tenellus*	*Psilocarphus*	Asteraceae	Yes	B
*Sanicula bipinnatifida*	*Sanicula*	Apiaceae	Yes	U
*Senecio vulgaris*	*Senecio*	Asteraceae	No	U,E
*Sidalcea calycosa*	*Sidalcea*	Malvaceae	Yes	E,B
*Trifolium depauparatum*	*Trifolium*	Fabaceae	Yes	E
*Trifolium microcephalum*	*Trifolium*	Fabaceae	Yes	U
*Trifolium variegatum*	*Trifolium*	Fabaceae	Yes	U,E,B
*Trifolium willdenovii*	*Trifolium*	Fabaceae	Yes	U
*Triphysaria eriantha*	*Triphysaria*	Orobanchaceae	Yes	E
*Triteleia hyacinthina*	*Triteleia*	Asparagaceae	Yes	U

*Note*: All species were identified during the flowering phenophase.

**TABLE 3:** Analysis of variance of Jaccard and Sorenson dissimilarity indices for each horizontal and vertical beta diversity class.

Level	Observations	Least Sq mean	SE	Mean	Tukey HSD
**Sorensen dissimilarity**					
H	27	0.22	0.02	0.22	B
HHT	54	0.28	0.02	0.28	B
HT	27	0.19	0.02	0.19	B
HV	54	0.76	0.02	0.76	A
V	27	0.72	0.02	0.72	A
VT	54	0.76	0.02	0.76	A
VVT	108	0.77	0.01	0.77	A
**Jaccard dissimilarity**					
H	27	0.36	0.02	0.36	C
HHT	54	0.42	0.01	0.42	C
HT	27	0.32	0.02	0.32	B
HV	54	0.86	0.01	0.86	A
V	27	0.83	0.02	0.83	A
VT	54	0.86	0.01	0.86	A
VVT	108	0.87	0.01	0.87	A

*Note*: The number of observations for each comparison class is the total number of comparisons that could be made. For instance, there are three zones across three pools and years, resulting in 27 observations. In contrast, there are three factorial pools, three factorial years, and three zones of vegetation that can be compared for VVT, resulting in 108 observations. A nonparametric post‐hoc Tukey significant honest difference estimate was calculated due to nonequal observation sizes.

**FIGURE 4:**
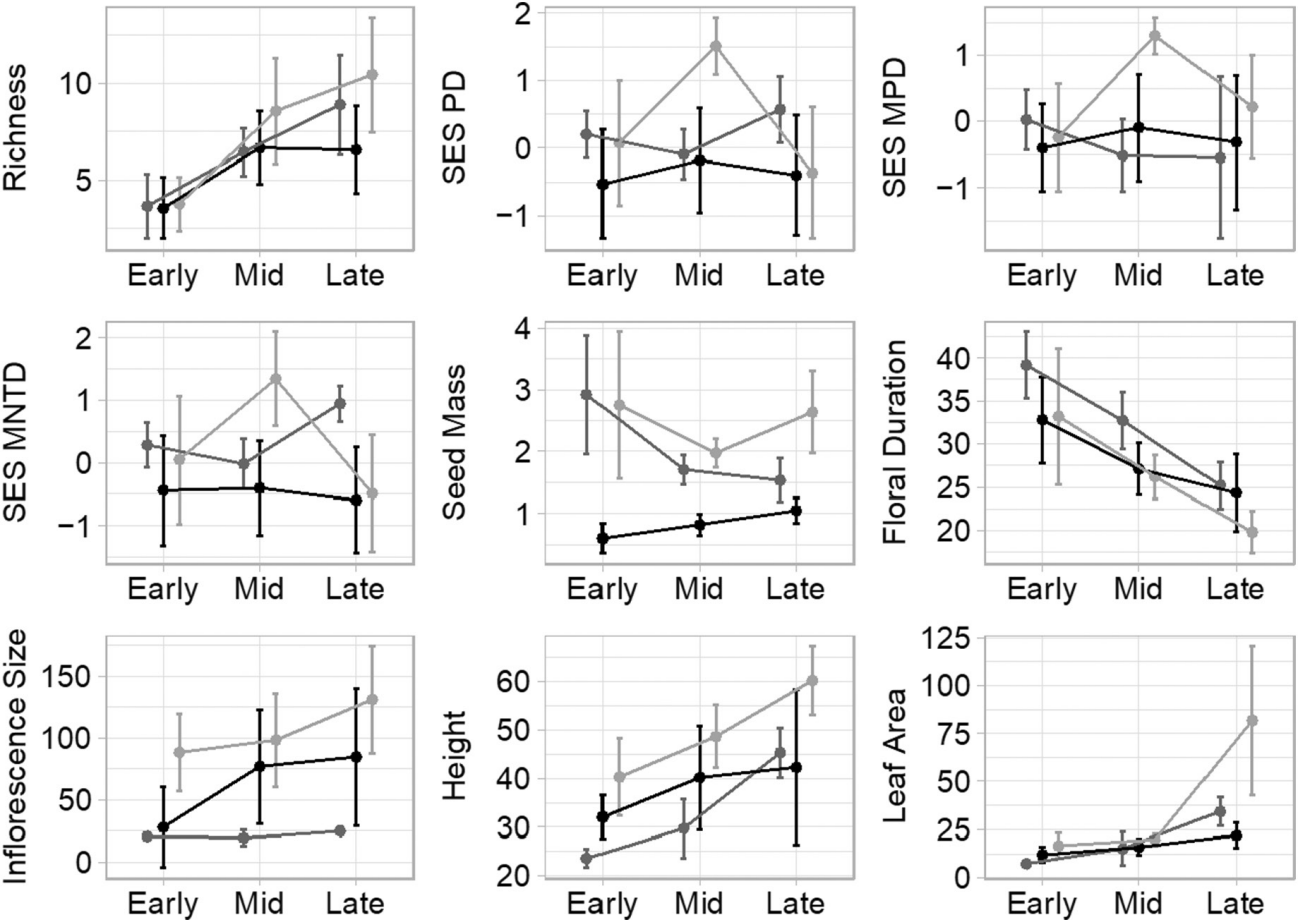
Change of average taxonomic, phylogenetic, and functional diversity between the early, mid, and late spring seasons for each vegetation zone. Taxonomic diversity is measured as the richness of flowering species found in each zone. Phylogenetic diversity was quantified as the standard effect size of Faith's phylogenetic diversity. Phylogenetic structure was measured as the standard effect size of mean nearest taxon distance (SES MNTD) and mean phylogenetic distance (SES MPD). In addition, community‐wide averages of five functional traits related to competition (leaf area & height), herbivore food availability (seed mass), and pollinator food availability (floral duration & inflorescence size) were recorded for each zone of vegetation community. Standard deviation is displayed as error bars for each zone of vegetation and season.

Taxonomic diversity of flowering plants for all zones rose across seasons (Figure 4). Alpha diversity in the upland and edge zones continuously rose from the early though late season. The pool bottom exhibited the greatest change in richness from the early to mid season followed by a slightly negative change in richness from the mid to the late season.

**FIGURE 5:**
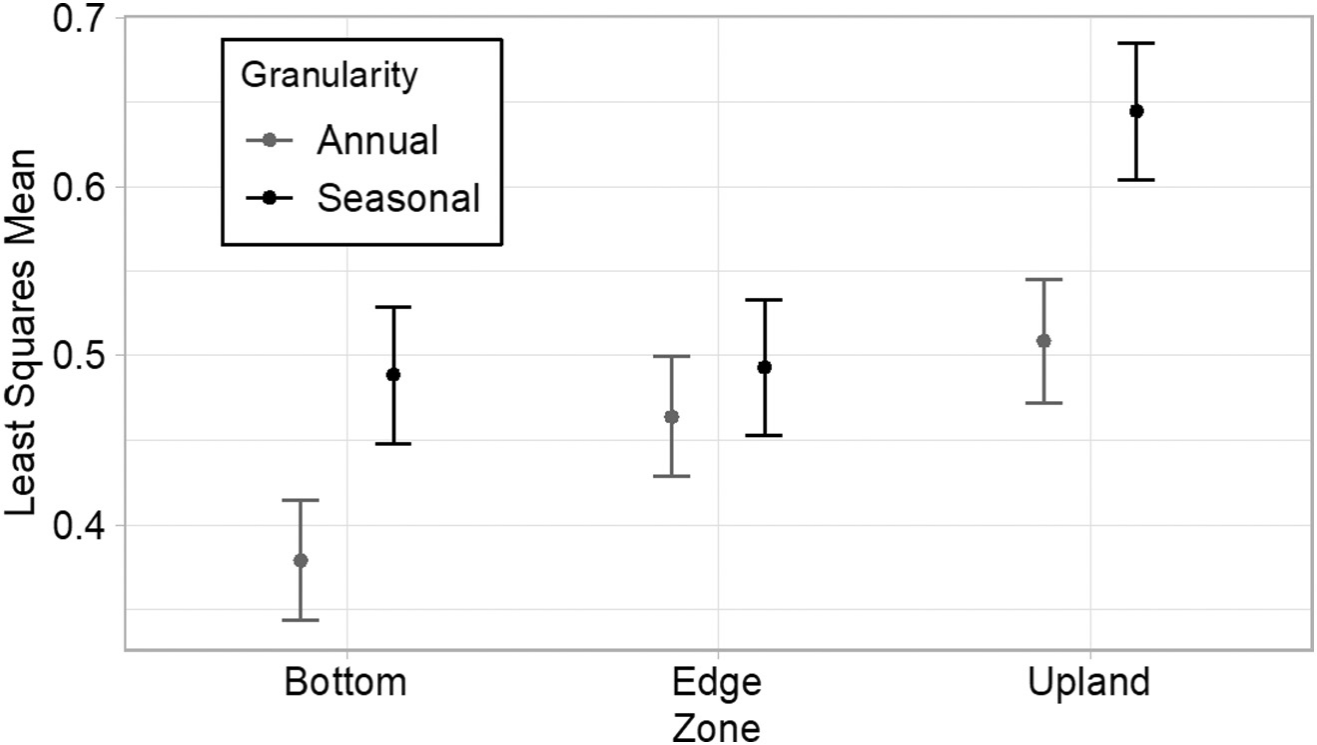
Least squares mean of beta diversity, measured as Jaccard dissimilarity, for each zone‐of‐vegetation between years (annual) and seasons. Jaccard dissimilarity was calculated for a single zone within a pool between years (ie. 2019, 2020, 2021) and seasons (ie. early, mid, and late).

Seasonal climate variation is the primary temporal driver of community turnover in vernal pool habitats. Although the alpha diversity within zones of vegetation differs little between years, annual and seasonal climate variation remain associated with significant changes in community composition (Table 4). Significant climate metrics are mean temperature, maximum temperature, and accumulated rainfall. Seasonal mean and maximum temperature variation were more associated with community turnover than interannual temperature variation. In contrast, interannual variation of precipitation was more strongly correlated with community turnover than seasonal precipitation change. Furthermore, a direct comparison of course‐grained temporal heterogeneity (i.e., interannual) and fine‐grained temporal heterogeneity (i.e., intraannual) across each zone found a significantly higher seasonal effect than annual effect on community turnover for the pool bottom and upland (Figure 5). Ultimately, zones of vegetation exhibit relatively stable communities across time though community turnover is influenced by seasonal temperature and interannual precipitation variability.

**TABLE 4:** Summary of mantel tests conducted on the variance of community turnover, phylogenetic diversity, MNTD, and MPD explained by several seasonal and annual environmental variables –maximum temperature, mean temperature, and precipitation.

Environmental variable		Community	Turnover	PD		MNTD		MPD	
		*z* Statistic	*p*‐Value	*z* Statistic	*p*‐Value	*z* Statistic	*p*‐Value	*z* Statistic	*p*‐Value
Pool Wide	Annual Max Temp	205.02	**.0190**	307.93	.3790	290.77	.5385	268.02	.8482
	Annual Mean temp	84.34	**.0110**	123.97	.9900	119.15	.9800	120.36	.0729
	Annual Precipitation	611.44	**.0229**	927.97	.3000	874.89	.5704	802.48	.9221
	Season Max Temp	1378.93	**.0010**	2242.45	**.0090**	1998.73	.1349	1744.96	.9620
	Season Mean Temp	1017.92	**.0010**	1551.86	**.0150**	1379.67	.2987	1208.69	.5345
	Season Precipitation	254.67	**.0050**	393.80	.0870	379.28	.0809	329.83	.7403
Bottom	Annual Max Temp	173.52	**.0170**	184.45	.4126	199.40	.7103	260.27	.8921
	Annual Mean temp	70.22	**.0230**	76.34	.4505	85.02	.3327	112.41	.9520
	Annual Precipitation	513.13	**.0210**	550.31	.4605	594.39	.9620	783.23	.6044
	Season Max Temp	1175.28	**.0010**	1138.62	.6683	1251.92	.6703	1694.37	.8901
	Season Mean Temp	875.60	**.0010**	869.21	.2368	992.52	.0549	1269.19	.6304
	Season Precipitation	216.37	**.0010**	226.38	.5944	246.42	.9341	333.45	.8601
Edge	Annual Max Temp	194.21	**.0130**	311.57	.6004	271.45	.6204	280.53	.5824
	Annual Mean temp	79.57	**.0010**	131.95	.3966	119.02	.2967	126.20	.0989
	Annual Precipitation	574.88	**.0130**	955.94	.3526	842.79	.4066	868.34	.4406
	Season Max Temp	1311.32	**.0010**	1986.36	.9081	1696.25	.9441	1783.32	.6983
	Season Mean Temp	945.32	**.0010**	1425.60	.9840	1245.10	.7762	1287.86	.6843
	Season Precipitation	239.79	**.0010**	378.51	.7143	329.17	.8661	333.84	.3926
Upland	Annual Max Temp	221.09	**.0050**	439.71	.4825	447.10	.0929	313.63	.5964
	Annual Mean temp	90.82	**.0070**	178.40	.9061	183.50	.0869	128.71	.5964
	Annual Precipitation	656.01	**.0090**	1329.10	.3626	1329.17	.0949	964.94	.4266
	Season Max Temp	1483.24	**.0010**	3078.97	.0709	2817.82	.1049	2247.48	**.0250**
	Season Mean Temp	1088.90	**.0010**	2195.74	**.0330**	2071.28	**.0190**	1530.65	.0989
	Season Precipitation	273.99	**.0050**	580.14	**.0450**	534.4	.0629	432.20	**.0110**

*Note*: Tests were performed using all species within a pool, whole pool, and or the communities of species found within the bottom, edge, and upland vegetation zones.

Bold values indicate the significant effect of an environmental predictor on community turnover and phylogenetic metrics.

### 3.2 Phylogenetic beta diversity

**FIGURE 6:**
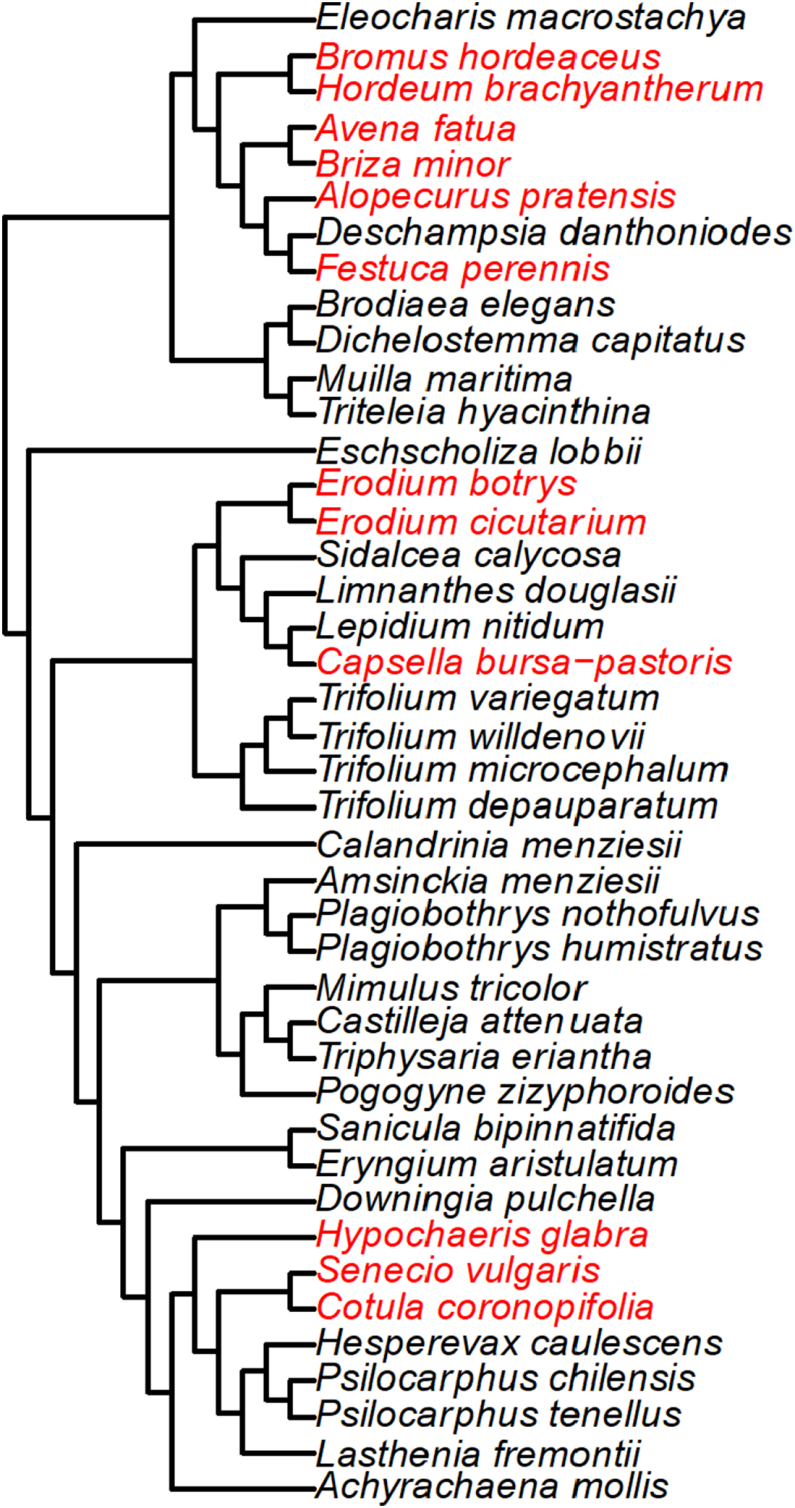
Phylogenetic tree of 42 vernal pool species (red: invasive, black: native) observed across three observation pools at the UC Merced, Grassland, and Vernal Pool reserve from 2019 to 2021 spring seasons. Phylogenetic relationships were determined using the Smith & Brown (2018) mega‐phylogeny and trimmed using V.PhyloMaker2 package in R.

**FIGURE 7:**
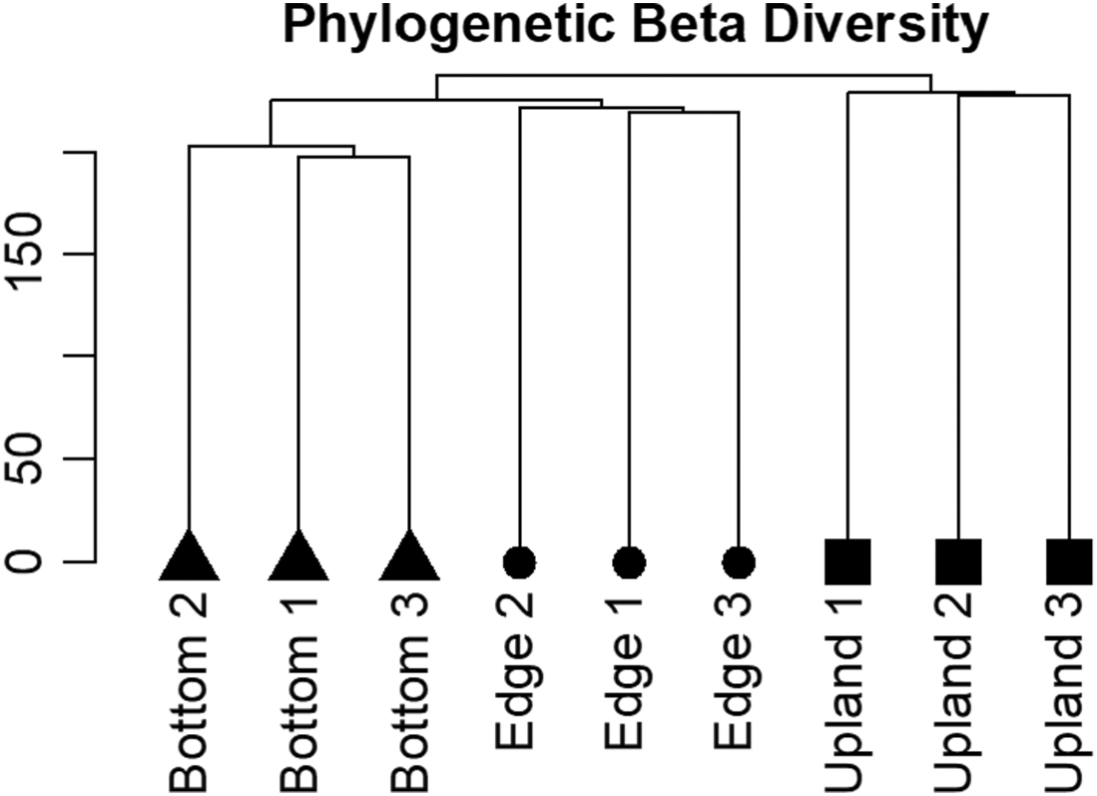
Clustered dendrogram of phylogenetic beta diversity across years for vegetation zones. Each observational community was composed of all species found within a vegetation zone for a given year.

A Synthesis phylogeny of 42 species was successfully constructed with no polytomies (Figure 6). Phylogenetic beta diversity was highest between zones and lowest across pools. Each zone was clustered into a unique monophyletic group (Figure 7), suggesting that zones‐of‐vegetation are phylogenetically unique units in the Merced vernal pool region.

**FIGURE 8:**
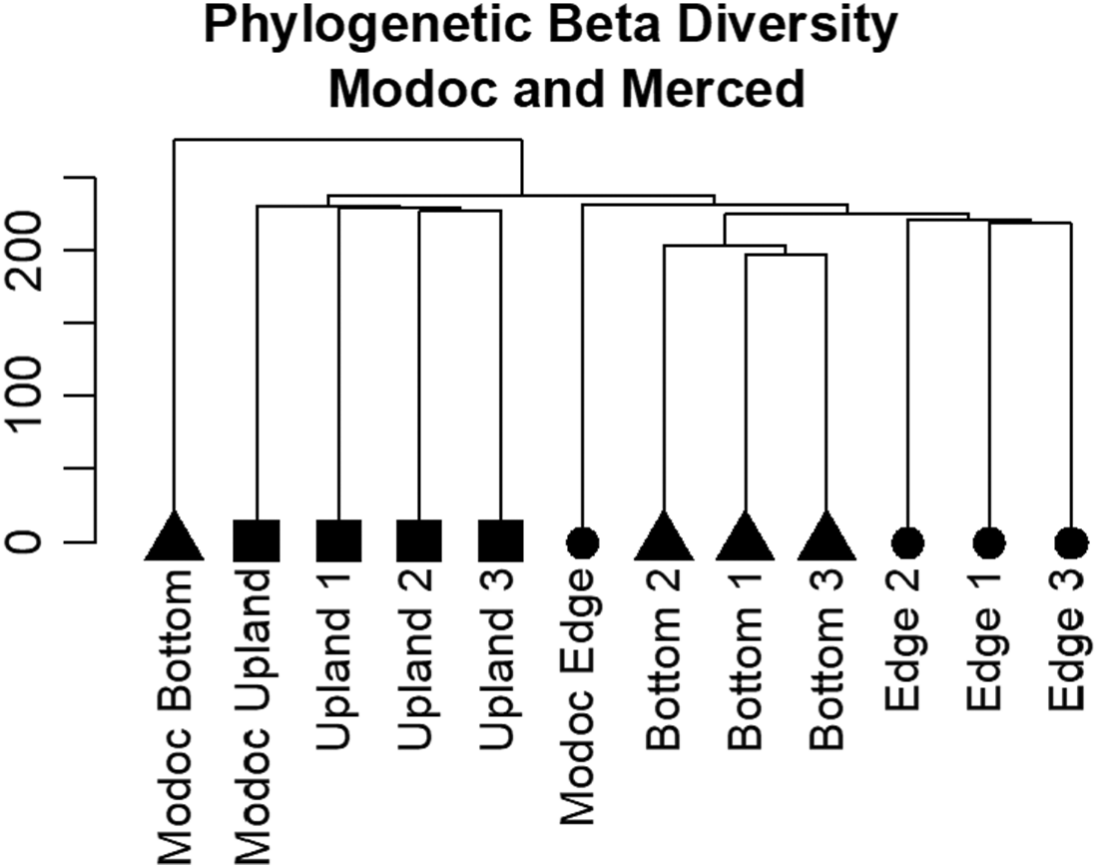
Phylogenetic beta diversity cluster dendrogram of community observations by zone for three Merced vernal pools and one vernal pool in Modoc, CA, described in Gosejohan et al. (2017).

When incorporating zones from the Modoc vernal pool complex, it was found that pool bottom communities are regionally distinct, whereas upland communities are regionally homogenous. The Modoc and Merced grassland communities are clustered together (Figure 8) suggesting similar phylogenetic diversity between regions. On the other hand, the pool bottom community of Modoc was distinct from the Merced bottom communities. Phylogenetic beta diversity analysis reveals that the Modoc pool bottom community is sister to all other zones‐of‐vegetation, and most phylogenetically distant from the Merced pool bottom communities (Figure 8).

### 3.3 Effect of inundation, invasion, and competition on the phylogenetic structure

Vernal pool bottom communities exhibited phylogenetic clustering tree wide (Table 5), suggesting that habitat filtering drives community structure in pool bottoms at the level of genera and families. Clustering persisted when invasive species were removed from the model (Table 5), indicating that native species adapted to predictable flooding as more closely related than by chance in the pool bottom. Uplands exhibited phylogenetic overdispersion tree wide (Table 5). When 9 invasive species – six Eurasian grasses within the Poaceae family and three nongrass species–were removed from the model, phylogenetic overdispersion was no longer found. There was no evidence of phylogenetic clustering or overdispersion in the edge community of the three observation pools.

**TABLE 5:** Standardized effect size of mean nearest taxon distance and mean phylogenetic distance for each zone‐of‐vegetation with or without invasive species included.

Clusters	Richness	SES MNTD	*p*‐Value	Tips	SES MPD	*p*‐Value	Tree wide
Bottom	16	−0.01	.51	Random	**−3.73**	**<.0001**	**Clustered**
Edge	14	−0.45	.33	Random	0.57	.69	Random
Upland	23	−0.91	.19	Random	**1.56**	**.96**	**Over‐Dispersed**
Bottom: NO INVASIVES	13	0.70	.75	Random	**−2.47**	**.02**	**Clustered**
Edge: NO INVASIVES	9	−0.22	.43	Random	−0.16	.36	Random
Upland: NO INVASIVES	14	0.39	.64	Random	0.17	.50	Random

*Note*: *p*‐Values are determined by comparing the observed SES distribution to a null community distribution with tip labels shuffled and permuted 999 times. SES MNTD is sensitive to structure at the tips of a phylogeny, such that clustering or overdispersion indicates that recently diverged species contribute to observed phylogenetic structure. SES MPD is more sensitive to structure that occur at deeper nodes of a phylogeny, such that observed phylogenetic structuring of a community is driven by niche overlap of taxonomic groups like genera or families.

Bold values indicate that SES MNTD and/or SES MPD *p*‐values were below 0.05 or above 0.95, which indicate that observed MPD and MNTD for each community is significantly different than the null distribution.

Interannual variation of inundation length leads to temporary instances of habitat filtering at the edge of a vernal pool. MNTD and MPD were both negatively correlated with inundation length in the edge community (Table 6). On the other hand, the pool bottom community was neither more nor less phylogenetically structured due to inundation length. These patterns suggest that interannually oscillating water levels affect communities partially composed of species not adapted to flooding, such as those found in the edge, whereas it has little effect on communities composed of species adapted to predictable flooding, as found in the bottom.

**TABLE 6:** Linear regression of inundation length and phylogenetic structure, measured as standard effect size of mean nearest taxon distance (SES MNTD) and mean phylogenetic distance (SES MPD), for the pool bottom and edge vegetation zones.

Phylogenetic variable	Zone	df	df error	*F* ratio	Estimate	SE	*p*‐Value	*R* ^2^
SES MNTD	Bottom	1	25	0.10	0.02	0.06	.76	.00
	**Edge**	**1**	**24**	**8.28**	**−0.35**	**0.12**	**.01**	**.26**
SES MPD	Bottom	1	25	0.01	−0.01	0.09	.92	.00
	**Edge**	**1**	**24**	**7.90**	**−0.34**	**0.12**	**.01**	**.25**

*Note*: The pool upland was excluded from the analysis because flooding does not occur in that vegetation zone. Observations were made for three pools over 3 years and three sub‐seasons (early, mid, and late spring seasons). Communities included in the calculation of SES MPD and SES MNTD had more than one species flowering at the time of observation.

Communities that exhibit significant associations betweeen the length of innundation and each phylogenetic variable are bolded.

Community‐wide average height and community‐wide average leaf area of the upland were negatively associated with MNTD, indicating greater phylogenetic clustering because of greater competitiveness (Table 7). In addition, community‐wide variance (CWV) was positively correlated with MNTD. This means that community‐wide mean of competitiveness was driven by the whole community of plants, not just an outlier with high‐trait values skewing the mean. When invasive plants were removed from the analysis, the relationship between competition and MNTD disappeared, suggesting that the invasive grasses were driving competition and phylogenetic clustering. Edge and pool bottom communities exhibited significant phylogenetic overdispersion in response to higher competition. This pattern did not disappear when invasive species were removed, suggesting the relationship was not driven by invasion but rather general instances of competitive exclusion of closely related species. Pool bottom communities had a significantly negative CWV in response to MNTD, whereas pool edge communities had parallel slopes of CWV and CWM in response to MNTD. The latter indicates that one or a few competitive species drive phylogenetic overdispersion.

**TABLE 7:** Competitiveness of a community by phylogenetic relatedness for each zone.

Phylogenetic variable	Zone	Response variable	df	df error	*F* ratio	Estimate	SE	*p*‐Value	*R* ^2^
SES MNTD	Bottom	PC Mean	1	25	21.39	1.73	0.37	<.0001	.46
	Bottom	PC Variance	1	25	5.57	−1.11	0.47	.0263	.18
	Edge	PC Mean	1	24	5.49	0.69	0.29	.0278	.19
	Edge	PC Variance	1	24	3.96	0.59	0.30	.0581	.14
	Upland	PC Mean	1	24	8.71	−0.59	0.20	.007	.27
	Upland	PC Variance	1	24	11.11	0.65	0.20	.0028	.32
SES MPD	Bottom	PC Mean	1	25	0.0003	0.006	0.34	.9864	.00
	Bottom	PC Variance	1	25	0.28	−0.18	0.35	.60	.01
	Edge	PC Mean	1	24	16.17	0.99	0.25	.0005	.40
	Edge	PC Variance	1	24	9.24	0.82	0.27	.0056	.28
	Upland	PC Mean	1	24	1.79	−0.39	0.29	.1933	.07
	Upland	PC Variance	1	24	11.68	0.86	0.25	.0023	.33

*Note*: Principle components of mean and variance were calculated using the community‐wide mean (CWM) and community‐wide variance (CWV), respectively, of leaf area and height. Each observation is a community recorded for each pool, each year, and each season, resulting in 27 total observations, however, observations with only one plant species could not be used to calculate SES MPD or SES MNTD, resulting in <27 observations for each model.

### 3.4 Climatic drivers of phylogenetic diversity

Phylogenetic diversity is driven primarily by seasonal climatic variation. Mantel tests found that seasonal maximum and mean temperature drive phylogenetic dissimilarity between whole pool communities, whereas seasonal precipitation and all annual climatic factors do not (Table 4). When analyzing zonal responses to seasonal and annual climatic change, the upland zone was the only community that showed significant responses to seasonal maximum temperature and seasonal precipitation. I find that phylogenetic diversity is less sensitive to seasonal and annual climate variance than taxonomic diversity.

**FIGURE 9:**
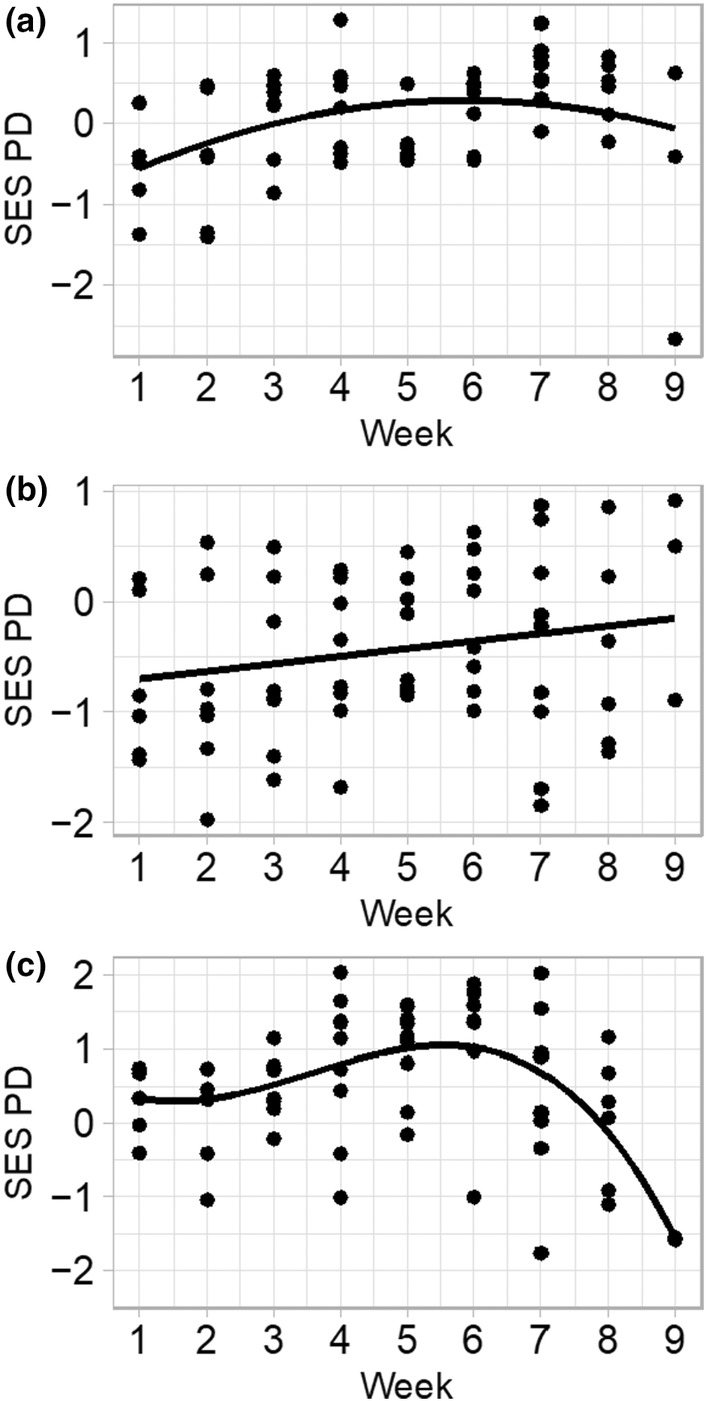
Standardized effect size of Faith's phylogenetic diversity (SES PD) by week for pool bottom (a), edge (b), and upland (c). Observations were made for each vegetation zone for three pools and 3 years. The best‐fit line is the best‐fitting polynomial regression determined using Akaike's information criterion. Shaded regions are standard errors for each model.

In general, MNTD and MPD exhibited no relationship with seasonality independent of zone (Table 8), whereas PD_Faith_ was significantly associated with seasonality. The edge and bottom community exhibited no change in either MNTD, MPD, or PD_Faith_ across seasons when analyzing the early, mid, and late season 3‐week time bins (Figure 4). On the other hand, the pool upland community had significantly elevated MNTD, MPD, and PD_Faith_ in the mid‐season compared to the early and late seasons, indicating a momentary instance of phylogenetic overdispersion in the upland. Polynomial regression models of weekly observations further support this conclusion in the upland, where the best fitting cubic regression model shows a steep drop off in PD_Faith_ starting in the sixth week and a trend towards phylogenetic clustering during the late growing season (Figure 9). All zones had negative phylogenetic diversity in the first 3 weeks, suggesting clustering in the early season, followed by a sharp rise in community phylogenetic diversity when seasonal conditions became mild.

**TABLE 8:** Multiple regression table of several phylogenetic diversity, functional diversity, and taxonomic diversity metrics by zone, season, and their interaction as predictor variables.

Response variable	Zone	Season	Zone * season	*R* ^2^	Observations	*F* ratio
Seed Mass	**<0.0001**	**0.00195**	**<0.0001**	.68	81	19.05
Inflorescence Size	**<0.0001**	**0.0018**	**0.085**	.59	81	13.03
Leaf Area	**<0.0001**	**<0.0001**	**<0.0001**	.72	81	23.26
Height	**<0.0001**	**<0.0001**	0.114	.64	81	15.76
Floral Duration	**<0.0001**	**<0.0001**	0.275	.66	81	17.56
SES PD_faith_	**0.0004**	**0.018**	**<0.0001**	.44	81	7.12
SES MNTD	**<0.0001**	0.14	**<0.0001**	.47	79	7.65
SES MPD	**0.0006**	0.0741	**0.0055**	.35	79	4.75
Richness	**0.0033**	**<0.0001**	0.085	.54	81	12.5

*Note*: Community‐wide mean (CWM) was calculated for each functional diversity metric by averaging the trait values of all species found within a community. There were 81 communities observed for three zones, in three pools, over 3 years and three seasons that SES PD, richness, and community‐wide averages of seed mass, inflorescence size, leaf area, height, and floral duration that could be used in multiple regressions with zone and season. There were only 79 communities with species richness greater than one that could be used to calculate SES MNTD and SES MPD used for subsequent multiple regressions.

*P*‐values are reported for each predictor variable, with significant associations with response variables indicated by bolded *p*‐values.

### 3.5 Seasonal and phylogenetic associations with functional diversity

Zones of vegetation exhibited significant differences in regards to functional traits: leaf size, height, floral duration, seed mass, and inflorescence size (Table 8). In addition, all functional traits changed significantly between season (Figure 4). Duration of flowering and seed mass were significantly greater in pool bottom plants and exhibited a steep decline from the early to late season. Leaf Area, inflorescence size, and height were all significantly greater in upland communities and rose significantly during the growing period.

Several traits exhibited strong associations with phylogenetic diversity, however, this was dependent upon the zone examined. All traits other than seed mass were significantly associated with phylogenetic diversity at the whole pool level (Table 9). Phylogenetic diversity was positively correlated with inflorescence size, leaf area, height, and seed mass in the edge community. Leaf area and height were positively associated with phylogenetic diversity in the pool bottom, while seed mass was negatively associated with phylogenetic diversity at the pool bottom and upland. In all zones, floral duration was negatively correlated with phylogenetic diversity. The predictive power of phylogenetic diversity on functional trait responses was relatively high, however, phylogenetic diversity cannot consistently predict the direction of functional traits in all community types.

**TABLE 9:** Linear regression summary of phylogenetic diversity and functional traits – seed mass, inflorescence size, leaf area, height, and floral duration – for whole pools and each vegetation zone.

Functional trait	Vegetation zone	df	Estimate	SE	*p* Value	*R* ^2^
Seed Mass	Whole Pool	79	−0.0006	0.0004	.1630	.02
	**Bottom**	**25**	**−0.003**	**0.0004**	**<.0001**	.77
	**Edge**	**25**	**0.0009**	**0.0002**	**<.0001**	.51
	**Upland**	**25**	**−0.002**	**0.0005**	**.0004**	.40
Inflorescence	**Whole Pool**	**79**	**0.08**	**0.02**	**.0005**	.14
Size	Bottom	25	0.01	0.00	.1250	.09
	**Edge**	**25**	**0.20**	**0.03**	**<.0001**	.67
	Upland	25	0.02	0.03	.5419	.02
Leaf Area	**Whole Pool**	**79**	**0.03**	**0.01**	**.0029**	.11
	**Bottom**	**25**	**0.04**	**0.01**	**.0002**	.43
	**Edge**	**25**	**0.02**	**0.00**	**.0001**	.45
	Upland	25	0.02	0.03	.5740	.01
Height	**Whole Pool**	**79**	**0.03**	**0.00**	**<.0001**	.36
	**Bottom**	**25**	**0.03**	**0.01**	**<.0001**	.54
	**Edge**	**25**	**0.04**	**0.01**	**<.0001**	.62
	Upland	25	0.01	0.01	.0555	.14
Floral	**Whole Pool**	**79**	**−0.02**	**0.00**	**<.0001**	.54
Duration	**Bottom**	**25**	**−0.03**	**0.00**	**<.0001**	.87
	**Edge**	**25**	**−0.02**	**0.00**	**<.0001**	.72
	**Upland**	**25**	**−0.02**	**0.00**	**.0001**	.45

*Note*: Functional traits were measured as community‐wide averages and phylogenetic diversity was measured as PD_Faith_.

Functional traits significantly associated with phylogenetic diversity are indicated by bolded values for each vegetation zone and the whole pool.

## 4 DISCUSSION

The novelty of this study is that ‘zones‐of‐vegetation’ are not random plant assemblages produced by dispersal limitation but unique and stable phylogenetic communities shaped by habitat conditions. Furthermore, invasion causes phylogenetically clustered communities due to the exclusion of native species and flooding causes phylogenetic clustered communities due to the exclusion of flood‐intolerant plant species. The two alternative hypotheses of competition's effect on phylogenetic structure are both found within the vernal pool habitat, though I find the outcome of competition is a consequence of whether the community is dominated by invasive or native species.

### 4.1 Taxonomically defined zones‐of‐vegetation

The composition of vernal pool communities greatly varies at different geographic scales. Climate and source biota change along latitude, resulting in different floristic communities at a regional scale (Holland, 1976; Holland & Dains, 1990). For instance, Buck (2004) found only 14% of species were shared between three vernal pool regions from Central to Northern California. As such, vast taxonomic differences between regions provides the impetus to characterize the species composition within the Merced vernal pool complex, which had not previously been published. Concerning the microhabitat, differences of precipitation year drive the length of inundation and influence the diversity of species and distribution within a pool (Bauder, 2000; Bliss & Zedler, 1998; Martin & Lathrop, 1986; Schlising & Sanders, 1982). Annual and seasonal climates were significantly correlated with community dissimilarity, suggesting that precipitation year does indeed drive community differences. However, I found that seasonal climatic variance dominates community turnover rates, which contrasts with previous climatic associations in vernal pools (Buck, 2004). Ultimately, community turnover rates within the three Merced observation pools are affected by local pool features and climate variability in line with general predictions about community turnover in other habitats.

Within a pool, clearly defined zones‐of‐vegetation that correspond with abiotic gradients were discovered. Many abiotic gradients exist along the slope of a pool – salinity, clay content, inundation length, and pH (Bliss & Zedler, 1998; Gerhardt & Collinge, 2003; Holland & Dains, 1990; Holland & Jain, 1988; Linhart, 1976)– that select for different vegetation communities (Faist & Collinge, 2015; Gerhardt & Collinge, 2007), though the primary driver of community composition was found to be inundation in natural vernal pools (Gosejohan et al., 2017). Time of inundation is important for controlling species distributions within a pool in accordance with species tolerances (Bauder, 2000; Emery et al., 2009; Holland & Jain, 1988; Schlising & Sanders, 1982), causing heterogeneous zones‐of‐vegetation along the slope of a pool (Barbour et al., 2003; Deil, 2005; Gosejohan et al., 2017; Solomeshch et al., 2007). In this study, I initially differentiated zones based on inundation, separating the upland from the edge and pool bottom and clay content, which differentiated the edge from the pool bottom. Using this abiotic heuristic, I found community dissimilarity is much higher between zones (vertical beta diversity) than between the same zone of two different pools (horizontal beta diversity), which strongly suggests that the zonal communities are defined by common traits and abiotic tolerance to flooding and/or clay content.

Furthermore, I found that a zone within the same pool will be 1. stable between years, conforming to previous predictions about interannual community turnover, and 2. will be less influenced by interannual climate variability than geographic distance. Buck (2004) found that floristic dissimilarities were largely driven by spatial variation rather than within‐year or between‐year environmental variation. These findings are similar to those of this study, in which H was significantly higher than HT, and HHT was significantly higher than H. A potential explanation for observed temporal stability is the presence of a highly diversified and persistent seed bank that is common to vernal pools (Bliss & Zedler, 1998), which has proven to be crucial for stabilizing community composition during times of high disturbance via rescue and storage effects (Chesson, 2000; Plue et al., 2020; Plue & Cousins, 2018; Royo & Ristau, 2013; Vandvik et al., 2016) as well as demographic buffering (Piessens et al., 2004).

### 4.2 Vernal pool phylogeny

The synthesis phylogeny of vernal pool species constructed in this study by pruning the Smith & Brown, 2018 megaphylogney of 75,000+ taxa has proven to be useful in teasing apart the evolutionary relationships between communities. All species recorded throughout the present study were within the Smith and Brown (2018) mega‐phylogeny, eliminating the need to append the synthesis phylogeny. The present synthesis phylogeny was not plagued by polytomies owing to the recent sampling of vernal pool species and extensive genetic work conducted by many researchers. Not too long ago, limited resolution of phylogenetic relationships among vernal pool species was commonplace. An angiosperm super‐tree produced by Davies et al. (2004) resulted in several polytomies among vernal pool Trifolium and Psilocharphus genera, and Asteraceae, Fabaceae, and Orobanchaceae families (Sargent et al., 2011). However, the same super‐tree produced highly resolved relationships among nonvernal pool species of the genera and families, highlighting the underrepresentation of vernal pool species in past databases. The success of vernal pool research is contingent upon the wealth or paucity of genomic information available, and the present paper is a testament to the recent and ongoing successes of research labs across the world.

### 4.3 Zones‐of‐vegetation as phylogenetic communities

I discovered that pool bottom, edge, and upland plant communities are unique phylogenetic communities. Phylogenetic beta diversity analyses determined that zones are more phylogenetically similar across moderate geographic distances than between two zones within the same pool. These findings suggest that diversification and habitat filtering at the local scale dominate phylogenetic patterns compared to ecological and evolutionary processes operating on the regional scale, such as dispersal limitation and priority effects. However, when including the Modoc Plateau vernal pool system, pool bottoms exhibited large phylogenetic differences between vernal pool regions. This finding is crucial to consider for conservational endeavors that aim not only to preserve a certain number of species but also to preserve lineages of ecological importance belonging to different regions. Although there were no replicate pools in Modoc these results suggest that vernal pool bottom communities are locally similar and regionally distinct in regard to phylogenetic diversity, highlighting the importance of treating zones‐of‐vegetation as unique communities within a pool and as unique communities between vernal pool regions. In contrast, uplands, which showed moderate levels of phylogenetic dissimilarity between pools in Merced, exhibited low‐phylogenetic distance from the Modoc pools. This similarity between distant regions is partially the result of similar invasive species occupying the pool uplands (8 invasive species shared between regions), highlighting the homogenizing effect of invasive species at a regional scale (Daru et al., 2021).

### 4.4 Ecological drivers of contemporary vernal pool communities

Vernal pool zones‐of‐vegetation exhibit significantly different phylogenetic patterns that point to the cause of community structuring in deep time and due to relatively recent human introductions. First, vernal pool bottom communities are phylogenetically clustered tree wide, a pattern that emerges due to severe environmental conditions that excludes nonadapted species (Cahill et al., 2008; Gerhold et al., 2015; Lososová et al., 2015; Price & Pärtel, 2013). Our findings suggest that inundation, which has long been associated with community structure in vernal pools, winnows the range of species to those that have homologous traits evolved from earlier transitions to environmental stressors present in vernal pool habitats. Empirical evidence of preconditioning to vernal pools of ancestral species has been found within the *Lasthenia* genera, in which a single ancestral species adapted to wetland environments resulted in multiple instances of independent evolution to vernal pools habitats (Emery et al., 2009). In addition, Tittes et al. (2019) discovered that all taxa belonging to the *Lasthenia* genera have the highest fitness under similar hydrologic conditions regardless of habitat affinity, suggesting homology for inundation tolerance. Such studies provide preliminary support for a connection between trait homology and plant distributions within a pool that, in the present study, form the foundation for the whole community phylogenetic patterns observed. I also find that the pool upland is phylogenetically overdispersed, largely owing to the introduction of Eurasian grass species that widens the number of evolutionarily distinct lineages found in the grassland. The pattern observed in the grassland of the Merced vernal pools is likely not generalizable to other habitats, especially habitats with relatively few invasive plants, rather, the present findings highlight that habitat level phylogenetic diversity can appear as overdispersed because of repeated invasions.

Second, the outcome of competition on phylogenetic structure is a consequence of invasion gradients, as competition from invasive plants produces phylogenetic clustering whereas competition between native plants produces phylogenetic overdispersion. The hypothesis that competitive groups will exclude noncompetitive groups leading to phylogenetic clustering has been weak (Gerhold et al., 2015; Lososová et al., 2016), whereas other studies find phylogenetic overdispersion due to competition (Pérez‐Toledo et al., 2022). The novelty of our findings is that phylogenetic structure is dependent upon the degree of invasion. The pool upland community exhibits strong phylogenetic clustering driven by competition between natives and closely related Eurasian grass species. When invasive species were removed from the MNTD analysis clustering disappeared. The invasive species recorded in this study were primarily from Europe, and common biogeographic and evolutionary history in highly competitive European grasses drove phylogenetic clustering observed in the upland. On the other hand, competitiveness was associated with phylogenetic overdispersion in the native rich communities of the pool bottom and edge, conforming to the ‘competitive relatedness hypothesis’ initially proposed by Darwin (1859) and later supported in various plant communities (Cavender‐Bares et al., 2004, 2006). Our findings provide further support that phylogenetic structure can be an outcome of human facilitated introductions (Castellani et al., 2022; Lishawa et al., 2019; Lososová et al., 2016).

### 4.5 Phylogenetic diversity between seasons

I discovered that phylogenetic diversity changes dramatically between seasons, supporting the hypothesis of this study, though the magnitude is contingent upon the zone under investigation. Previous studies of phylogenetic diversity through time examined PD patterns between discrete time units (Strauß et al., 2016), whereas no study has examined phylogenetic diversity over continuous time, such as on the scale of days or weeks. Using both continuous and discrete time, it was found that the pool upland exhibited significantly elevated phylogenetic diversity (MNTD, MPD, and PD_Faith_) in the mid‐season. This period corresponds with the transition from inclement weather to warmer, dryer spring conditions. Following the mid‐season, PD trends towards clustering. Invasive grass species began flowering in the latter half of the growing season, which likely drove the phylogenetic clustering observed in the upland during that period. An alternative explanation is climate, in which drought on drylands has been observed to cause shifts in community composition and promote diversity losses (Harrison et al., 2015; Lloret et al., 2009). In the pool bottom and edge community, phylogenetic clustering was observed during the early season, which coincides with the period of inundation. In previous studies, experimental reductions of rainfall resulted in opposing patterns of taxonomic and phylogenetic diversity, with taxonomic diversity falling in response to reduce rainfall and phylogenetic diversity rising (López‐Rubio et al., 2022). These patterns parallel the responses of PD to inundation in the edge community, in which inundation drove phylogenetic clustering, though I found that taxonomic species richness throughout the zones of a pool rose as soil moisture fell, contrasting with previous findings. For all zones, it was found that the early season was associated with phylogenetic clustering caused by habitat filtering from stressful flooded conditions. While not studied in this article, drought conditions in the latter half of the growing season may have promoted phylogenetic clustering in the upland. These patterns support the hypotheses that phylogenetic diversity fluctuates between seasons and that phylogenetic clustering caused by flooding is temporally dependent.

The findings of this paper suggest that competition by invasive species promotes phylogenetic clustering, at least when invasives originate from the same continent, and that competition between native species promotes phylogenetic overdispersion. Vernal pool habitats have taxonomically and phylogenetically distinct communities that exhibit unique phylogenetic responses to seasonal conditions. The community composition of vernal pool habitats is driven by adaptations to flooding and responses to invasion that has in turn translated into persistent phylogenetic structure.

## AUTHOR CONTRIBUTIONS

**Brandon Hendrickson:** Conceptualization (equal); data curation (equal); formal analysis (equal); investigation (equal); methodology (equal); project administration (equal); validation (equal); visualization (equal); writing – original draft (equal); writing – review and editing (equal).

## CONFLICT OF INTEREST STATEMENT

There are no conflicts of interest in the procurement of data or production of results.

### OPEN RESEARCH BADGES

This article has earned Open Data and Open Materials badges. Data and materials are available at 10.6084/m9.figshare.25823065; https://github.com/Brandon‐Thomas‐Hendrickson/Vernal_Pool_CommunityDiversity.git.

## Data Availability

All datasets are available on figtree (DOI: 10.6084/m9.figshare.25823065) and code used in this paper are available at https://github.com/Brandon‐Thomas‐Hendrickson/Vernal_Pool_CommunityDiversity.git.
